# Research on the structure function recognition of PLOS

**DOI:** 10.3389/frai.2024.1254671

**Published:** 2024-01-24

**Authors:** Jiangfeng Liu, Zhixiao Zhao, Na Wu, Xiyu Wang

**Affiliations:** ^1^School of Information Management, Nanjing University, Nanjing, China; ^2^Laboratory of Data Intelligence and Cross Innovation, Nanjing University, Nanjing, China; ^3^College of Information Management, Nanjing Agricultural University, Nanjing, China; ^4^Research Center for Humanities and Social Computing, Nanjing Agricultural University, Nanjing, China

**Keywords:** deep learning, structure function recognition, PLOS, BERT, informetrics

## Abstract

**Purpose:**

The present study explores and investigates the efficiency of deep learning models in identifying discourse structure and functional features and explores the potential application of natural language processing (NLP) techniques in text mining, information measurement, and scientific communication.

**Method:**

The PLOS literature series has been utilized to obtain full-text data, and four deep learning models, including BERT, RoBERTa, SciBERT, and SsciBERT, have been employed for structure-function recognition.

**Result:**

The experimental findings reveal that the SciBERT model performs outstandingly, surpassing the other models, with an F1 score. Additionally, the performance of different paragraph structures has been analyzed, and it has been found that the model performs well in paragraphs such as method and result.

**Conclusion:**

The study's outcomes suggest that deep learning models can recognize the structure and functional elements at the discourse level, particularly for scientific literature, where the SciBERT model performs remarkably. Moreover, the NLP techniques have extensive prospects in various fields, including text mining, information measurement, and scientific communication. By automatically parsing and identifying structural and functional information in text, the efficiency of literature management and retrieval can be improved, thereby expediting scientific research progress. Therefore, deep learning and NLP technologies hold significant value in scientific research.

## 1 Introduction

With the continual advancement of technology, the promotion of open access and developments in information technology have made scientific communication and collaboration more integrated and convenient. The number of academic papers is rapidly increasing. However, faced with an immense volume of literature resources, the need for researchers to quickly obtain the required information has become increasingly urgent. Research on structural and functional recognition in academic papers is fundamental. Academic papers' structural and functional aspects refer to the semantic description and summary of their internal structure, typically including sections like abstracts, introductions, methods, experiments, results, and conclusions. These aspects are crucial in scholarly articles, reflecting robust functionality and purpose. They provide readers with an overview of the article's content, aiding in quickly comprehending the literature's theme and scope.

However, manually identifying and extracting these structural and functional features can be time-consuming and labor-intensive. Deep learning technology has demonstrated promising results in natural language processing tasks, including structural and functional recognition, in recent years. Automating the recognition of these features can help researchers locate required information more swiftly and enhance work efficiency.

Specifically, with the support of artificial intelligence technology, significant advancements have been made in structural and functional recognition, especially in academic literature management. For instance, this technology can automatically extract structural and functional information, assisting users in better categorizing and searching the literature, significantly improving researchers' efficiency and work quality, and providing robust support for literature retrieval and knowledge management. Besides its application in academic literature management, structural and functional recognition technology finds applications in other areas, such as social media, e-commerce, and administrative management, helping users and government officials quickly identify specific information, thereby improving efficiency.

Although structural and functional recognition technology has many potential applications in scientific research, some challenges and issues remain. For example, in different academic disciplines, there may be significant differences in the structure and function of academic papers. Accurately identifying different types of structures and functions is still an urgent issue. In addition, without sufficient data and algorithm support for training and optimization of structural and functional recognition technology, it may lead to enough liability of the system, thereby affecting the work efficiency of researchers.

The Public Library of Science (PLOS) series of journals is a group of scientific journals characterized by open access. With high quality, high impact, and sustainability as their goals, they are committed to promoting the sharing and exchange of scientific knowledge. Studying the structural and functional recognition of PLOS series journals is significant. Firstly, structural and functional recognition can help us better understand the composition and operation of PLOS series journals. By identifying the various components of the journals and their relationships, we can gain a deeper understanding of the editorial policies, manuscript inclusion criteria, peer review processes, and other aspects of the journals, thereby improving our knowledge and mastery of the journal system. Secondly, structural and functional recognition can provide scientists with more effective publishing channels and communication platforms. Identifying the core journals in different fields and topics in the PLOS series can help scientists choose the appropriate journals for publishing their research results, share their research results on the journals, communicate and collaborate with peers, and promote further accumulation and dissemination of scientific knowledge. Finally, structural and functional recognition can also help evaluate the academic impact and quality of the PLOS series journals. By identifying the core content, highly cited articles, and authors of the journals, the status and value of the journals in the scientific community can be more accurately assessed, providing a more objective and scientific basis for scientists to choose appropriate publishing channels.

This paper proposes a deep learning-driven method to recognize the structural and functional components in the full-text articles of PLOS series journals. Data from PLOS series articles were collected, and the full text in XML format was preprocessed to extract the structural names of each paragraph and standardized. Multiple deep-learning models were then trained to recognize the structural and functional components in the text, and the models' performance was evaluated. Finally, this paper analyzed the language features of different structural and functional pieces.

## 2 Review

The research on structure-function recognition of academic literature refers to analyzing its structure and semantic content to better understand and extract information from it. This area of research mainly focuses on identifying different levels of academic papers and exploring the applicability of model algorithms from the perspective of disciplinary differences. These studies help achieve a fine-grained functional understanding of scientific literature abstract text. The analysis of literature structure can serve in the in-depth mining of scientific literature and knowledge discovery based on literature.

The research on the structure and function of academic texts has received attention from many scholars. Noriko proposed Background, Reference to the Previous Research, Purpose, Methods, Results, and Discussion (Kando, 1997). The typical article structure of Conclusion is predictable, which is beneficial for manually annotating the structure of academic papers at a fine-grained level and applying it. In the retrieval task, the experimental results showed that using text structure for full-text retrieval can achieve higher accuracy. Santiago Count 40 articles Exploring the structure and function of research papers in computer science. Currently, most documents in the introduction and discussion section align with the opening of IMRaD (Posteguillo, 1999).

The beginning and ending parts, with most remaining parts being model, algorithm analysis, etc. The rest can be classified as Methods and Results. Budsaba uses coding methods for biochemistry to research rhetorical devices in IMRaD structures to assist in reading and understanding better academic papers' research content (Kanoksilapatham, 2005). These studies are within a certain specific. The structure of a research paper is usually revealed in the introduction and method. This organizes the logic of experiments, results, and conclusions.

The automatic division of academic texts into IMRaD structures can be traced back to 2003. McKnight discovered sentences in abstracts of medical papers (McKnight and Srinivasan, 2003). The distribution of children follows the IMRaD structure, taking the positional information of the sentence as features and using supervised machine learning models to recognize sentences in abstracts. Minute built an automatic recognition system, and the sentence types were divided into five categories: Background Purpose, Methods, Results, and Conclusions (Mizuta et al., 2006). The best F1 value for Methods, Results, and Conclusions is 89.8%. Agarwal, it is believed that dividing sentences into IMRaD structures would be beneficial. They used polynomial Bayes for tasks such as automatic summarization and experimental result extraction models for functional delineation of sentences in biomedical papers. Fen has achieved good results among numerous multipliers (Agarwal and Yu, 2009). Ribeiro uses traditional machine learning classifiers for chapter functionality in academic papers. The recognition effect needs further improvement (Ribeiro et al., 2018). Lu et al. (2014) used vocabulary and sequence annotation methods to identify the structural functions of chapters, but however process for identifying titles containing unknown words still needs to be improved. Bowen Ma shows that the title of a chapter is more beneficial to the identification of the structure-function of academic documents than the in-chapter texts (Ma et al., 2021). Bowen Ma finds that compared with the chapter content; the chapter title is more conducive to identifying the structure of academic articles (Ma et al., 2022). Li et al. (2019) finds that disciplinary differences have a significant impact on the effectiveness of machine learning, with the recognition efficiency of the structure and function of academic texts in the medical field being significantly higher than that of other disciplines and the machine learning recognition effect of Methods and Results in common academic text functional structure frameworks is better.

## 3 Methods

### 3.1 Research process

**Figure 1:**
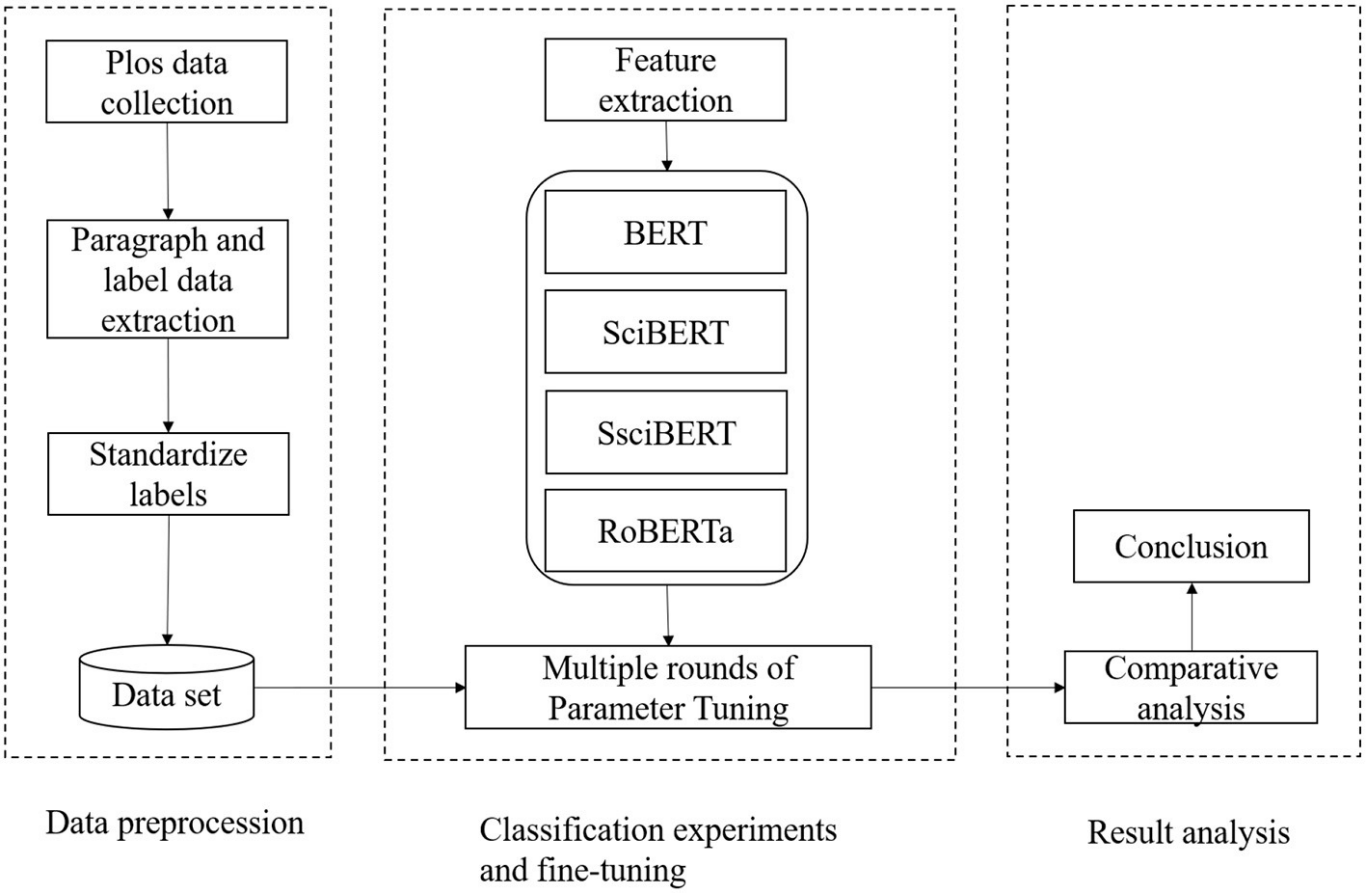
Research framework.

The basic process of using the deep learning model for paragraph-level structural and functional recognition is as follows. First, preprocess the input data and tokenize the paragraphs. Then, feed the tokenized sections into the model to obtain contextualized embeddings. After that, use the embeddings to train a classification model for paragraph-level structural and functional recognition. Finally, evaluate the model's performance on a test set and fine-tune it if necessary. This approach has been used successfully in various studies and has shown promising results in improving the efficiency of literature reviews and facilitating scientific communication. The overall framework of the study is shown in Figure 1.

### 3.2 Deep pre-trained language models

#### 3.2.1 Bidirectional encoder representation from transformers

Bidirectional Encoder Representation from Transformers (BERT) is a language representation model based on deep neural networks proposed by Google AI in 2018 (Devlin et al., 2018). It achieved the best performance in 11 different natural language processing tasks at its release. The main structure of the BERT model is composed of Encoders in the Transformer architecture. The BERT-base model uses 12 layers of Transformer Encoders as the neural network layer of the model.

A multi-layer perceptron classifier is added after the sentence representation output layer of the BERT model, and the Softmax activation function is used to classify the sentence at the output layer. The multi-layer perceptron consists of a fully connected layer and an output layer. The loss function of the output layer uses the cross-entropy loss function. Similarly, adding a fully connected layer on the last output layer of the BERT model can map the encoding vector output by BERT to the label set. Then, the Softmax activation function processes each token's output vector, and the numerical value of each dimension can represent the probability of the corresponding receipt for each type label.

BERT has many applications, including sentiment analysis, question-answering systems, automatic summarization, and more.

#### 3.2.2 SciBERT

SciBERT is a pre-trained language model based on the BERT structure designed specifically for scientific papers. Scientific texts have more domain-specific language structures and technical terminologies than general texts (Beltagy et al., 2019). SciBERT uses a custom vocabulary consisting of scientific paper terms called SCIVOCAB, which only has a 42% overlap with BASEVOCAB, the language used in BERT. This suggests significant differences between scientific and general texts, and the model can better handle domain-specific terminologies, such as technical jargon and proper nouns.

SciBERT was pre-trained on a large-scale corpus of biomedical and computer science research papers and exhibited superior performance on natural language processing tasks in these domains. In addition to these areas, SciBERT can be used in various applications, including semantic search, named entity recognition, and other scientific text-processing tasks. By leveraging the specialized vocabulary of scientific papers, SciBERT can accurately capture the context and meaning of technical terms, which is critical in scientific research and related applications.

#### 3.2.3 SsciBERT

SsciBERT is a BERT-based pre-trained language model adapted for social and humanities disciplines (Shen et al., 2023). It uses a corpus that includes a massive collection of abstracts from Social Sciences Citation Index (SSCI) journals. It conducts domain-adaptive pre-training on top of the BERT and SciBERT models. The relevant corpus covers research literature from 1986 to 2021. This model can be understood as further refining the BERT and SciBERT models. SsciBERT has shown higher accuracy than BERT and SciBERT in the prediction tasks of the language model, and there is a clear gap between the baseline model of BERT and SsciBERT. SsciBERT also achieved discipline classification, knowledge entity recognition, and sentence classification for social and humanities texts.

Furthermore, SsciBERT has a more extensive vocabulary related to the social and humanities disciplines, making it more capable of capturing these fields' nuances and technical terms. In addition, SsciBERT's fine-tuning on a large-scale corpus of SSCI abstracts has allowed it to understand better the specific language structures and patterns of the social and humanities fields. This makes it an ideal tool for applications such as social and humanities literature analysis, topic modeling, and sentiment analysis. Overall, SsciBERT represents a significant advancement in natural language processing for social and humanities disciplines.

#### 3.2.4 RoBERTa

RoBERTa is an optimized version of the BERT model that is more robust. It was developed by the Facebook AI research team and released in 2019 (Liu et al., 2019). Compared to BERT, RoBERTa removes the Next Sentence Prediction (NSP) task in its pre-training strategy and uses a character and word mixed text encoding method based on Byte-Pair Encoding (BPE). It also uses larger batch sizes, longer training times, and larger corpora during pre-training, which makes it perform better on multiple natural language processing tasks.

The RoBERTa model has high prediction accuracy and generalization ability, making it suitable for various natural language processing tasks such as text classification, named entity recognition, and question-answering systems. Its main advantage lies in the large amount of unannotated data used during pre-training, which enables the model to learn language representations that typically require a large amount of annotated data. RoBERTa utilizes dynamic masking to increase the model's robustness and generalization ability. This technique randomly masks some words from the input sequence, which ensures that the model is not dependent on any specific word order or position.

Moreover, the RoBERTa model has achieved state-of-the-art results on several natural language processing benchmarks, including GLUE, SuperGLUE, and SQuAD v2.0. It has also been used in real-world applications such as chatbots, language translation, and sentiment analysis. The success of RoBERTa has inspired many researchers to develop more advanced language models, such as GPT-3 and T5, which have achieved even more impressive results in natural language processing tasks.

### 3.3 Structural functional recognition task based on BERT

The BERT model is different from traditional sequential neural networks in that it uses a Transformer as a feature extractor to extract features from literature, which can more fully describe the word level, word level, and sentence level features of the paper and achieve recognition of the structure and function of the literature based on text classification. Firstly, input the literature alphabetically into the model and vectorize the words to obtain the word vectors (E1, E2,..., EN). Afterward, a multi-layer Transformer extracts features from the literature and generates feature vectors (T1, T2,..., TN). Finally, CRF is used to sequence constrain the output feature vectors to obtain the final recognition result. Among them, BERT's prediction layer adopts linear, fully connected, and Softmax normalization, and the classification task can modify the prediction.

## 4 Results

### 4.1 Data

This study obtained the complete PLOS series of academic papers from the PLOS website, which were saved in XML format. The data consisted of 336,168 files with a total size of 7.49 GB. Specifically, a customized program was developed to download the XML data of the documents from the PLOS website using web crawling technology and save it locally. Then, the paper data was parsed, and the required information, such as the article's title, abstract, author, and paragraph structure, was extracted using Python's XML parsing library. Subsequently, structural names were standardized using regular expressions and other methods to facilitate subsequent analysis and modeling. Finally, the processed data was stored in a database for following deep learning model training and experimentation. This data preprocessing is crucial as it can provide high-quality training data for following deep-learning models. The normalization process during preprocessing can reduce noise and uncertainty in the deep learning models, improving their accuracy and stability. Furthermore, data preprocessing can also help researchers better understand and explore text data to discover potential patterns and trends (Table 1).

**Table 1:** Normalization of structure-function names.

**Standard structure function name**	**Origin structure function name**
Introduction	Introduction, background, overview, summary, summary, and outlook
Materials and methods	Methods, material, and methods, models, models and methods, experimental procedures, design and implementation
Results	Results, results and discussion, model and results, experiments and results
Conclusion	Conclusion, conclusions and future directions, conclusions, and recommendations, findings, concluding remarks, availability, and future directions

### 4.2 Evaluation criteria

The Confusion Matrix is a standard matrix used in machine learning to visualize and calculate the performance of an algorithm. It is an efficient classification problem. Table 2 shows an example of a confusion matrix for a binary classification problem.


(1) $$\begin{array}{l} \text{P}=\frac{\text{TP}}{\text{TP}+\text{FP}}\;*\;100\% \end{array}$$

(2) $$\begin{array}{l} \text{R}=\frac{\text{TP}}{\text{TP}+\text{FN}}\;*\;100\% \end{array}$$

(3) $$\begin{array}{l} \text{F}1=\frac{2\;*\;{\text{P}}^{*}\text{ R}}{\text{P}+\text{R}}\;*\;100\% \end{array}$$

(4) $$\begin{array}{l} Macro\text{\_}P=\frac{1}{n}\overset{n}{\underset{i=1}{\sum }}P_{i} \end{array}$$

(5) $$\begin{array}{l} Macro\text{\_}R=\frac{1}{n}\overset{n}{\underset{i=1}{\sum }}R_{i} \end{array}$$

(6) $$\begin{array}{l} Macro\text{\_}F1=\frac{1}{n}\overset{n}{\underset{i=1}{\sum }}{F1}_{i} \end{array}$$

(7) $$\begin{array}{l} Weighted\text{\_}P=\overset{n}{\underset{i=1}{\sum }}P_{i}\;*\;f_{i} \end{array}$$

(8) $$\begin{array}{l} Weighted\text{\_}R=\overset{n}{\underset{i=1}{\sum }}R_{i}\;*\;f_{i} \end{array}$$

(9) $$\begin{array}{l} Weighted\text{\_}F1=\overset{n}{\underset{i=1}{\sum }}{F1}_{i}\;*\;f_{i} \end{array}$$

**Table 2:** Dichotomous confusion matrix.

**Truth**	**Forecast**	
	**Positive**	**Negative**
Positive	True positive (TP)	False negative (FN)
Negative	False positive (FP)	True negative (TN)

Specifically, precision (P) is used to measure the precision of the model, where TP represents the number of correctly recognized entities or sentences, and TP+TN represents the sum of the number of correctly identified entities (or sentences) and the number of incorrectly identified entities (or sentences). The recall is used to measure the recall of the model, where TP represents the number of correctly recognized entities or penalties, and TP+TN represents the sum of the number of correctly identified entities (or sentences) and the number of entities (or sentences) that have not been recognized (Equations 1–3).

The F1 score is a comprehensive evaluation index that considers both precision and recall and is a particular form of the F-beta score (see Equation 10), where β is a harmonic coefficient used to adjust the weight of precision P and recall R. The importance of precision P and recall R may vary in different natural language processing tasks. In this study, we believe they are equally essential and set the value of β to 1. The *F*-value, in this case, is generally called the F1 score (Equation 10).


(10) $$\begin{array}{l} F_{\beta }=\frac{\left(\beta^{2}+1\right)P*R}{\beta^{2}*\left(P+R\right)} \end{array}$$

For a single-label multi-class problem, a comprehensive metric is needed to measure the overall recognition performance of the model for all types. Macro Average and Weighted Average are standard metrics used to address this issue. In the Macro Average metric, each class's P, R, and F1 are first calculated separately, and then their arithmetic averages are computed to obtain Macro P, Macro R, and Macro F1 (Equations 4–6). Under the Macro Average metric system, all classes are assigned equal weights regardless of the number of instances, making it more suitable for evaluating tasks requiring consideration of all classes' performance. However, this evaluation can only reflect the model's actual performance when the data class distribution is balanced. In contrast, the Weighted Average first calculates each class's P, R, and F1 separately (Equations 7–9). Then, Weighted P, R, and Weighted F1 are calculated based on each class's actual number in the validation set. This method considers the weight relationship between different types and can better reflect the actual situation in the case of imbalanced data.

### 4.3 Experimental environment and hyperparameters

Due to the high-dimensional matrix operations required by deep learning models, more than personal laptops are needed to meet the experimental requirements. Therefore, all experiments in this study were conducted using high-performance GPU servers. The specific configuration of the server is as follows: GPU: 6 NVIDIA Tesla P40 graphics processors; CPU: 48 Intel (R) Xeon (R) CPU E5-2650 v4 @ 2.20GHz; Memory: 256GB; Video Memory: 24GB ^*^ 6 GPUs; Operating system: CentOS 3.10.0. Considering the recommended default parameter values for the models and the actual situation of the dataset, we conducted multiple experiments by comparing and adjusting the parameters. Finally, we selected the model parameter values, as shown in Table 3.

**Table 3:** Hyperparameters of deep pre-trained language models for sentence classification tasks.

**Parameter**	**Value**	**Description**
train_batch_size	16	Batch size for training
epochs	5	Number of training epochs
learning_rate	2e-5	The learning rate for training
max_sequence_length	128	Maximum sequence length for input tokens
test_batch_size	64	Batch size for testing

### 4.4 Experimental results of structure and function identification

Based on the F1 scores predicted by the four models in the above table, it is evident that SciBERT and SsciBERT outperform the BERT and RoBERTa models in all categories, with SciBERT performing the best. SsciBERT performs slightly less than SciBERT, but the difference is minimal. Looking at the four text categories, the models' performance could be better in Conclusion, possibly due to an imbalanced training dataset where Conclusion sentences have fewer instances relative to the other text categories. Furthermore, the models' classification performance is best in Materials and Methods, indicating that this text feature is more distinct than the different categories.

The superiority of SciBERT and SsciBERT over BERT and RoBERTa in predicting the text categories suggests that the former models are better suited for natural language processing tasks that require domain-specific knowledge. This could be because SciBERT and SsciBERT are trained in scientific texts and, as such, have a better understanding of scientific language than BERT and RoBERTa, which are trained in general readers. Moreover, the slight difference in performance between SciBERT and SsciBERT indicates that they are equally effective in classifying scientific texts.

However, these models' poor performance, in Conclusion, suggests that there may be a need for a more balanced training dataset that includes more Conclusion sentences. The conclusion is a crucial part of scientific papers, and accurate classification of this category is necessary for effective literature reviews and research analysis.

In summary, SciBERT and SsciBERT's models better predict text categories than BERT and RoBERTa. Although the performance difference is minimal between SciBERT and SsciBERT, they outperform BERT and RoBERTa in all types, especially in Materials and Methods. In Conclusion, the inferior performance of the models calls for more balanced training data to improve the classification of this category in scientific texts (Table 4).

**Table 4:** F1-score of the prediction results of different models.

**Model**	**BERT**	**RoBERTa**	**SciBERT**	**SsciBERT**
Introduction	0.87	0.88	0.90	0.89
Materials and Methods	0.90	0.90	0.92	0.92
Results	0.83	0.84	0.87	0.86
Conclusion	0.52	0.58	0.64	0.61
Accuracy	0.86	0.87	0.89	0.88
Macro avg	0.78	0.80	0.83	0.82
Weighted avg	0.86	0.87	0.89	0.88

## 5 Discussion

In recent years, the rapid development of deep learning has brought significant breakthroughs in various fields, including natural language processing (NLP). This study explored the potential of deep learning models for paragraph-level structure-function recognition using the full-text data of PLOS journals. We evaluated four different models, including BERT, RoBERTa, SciBERT, and SsciBERT, and compared their performance. Our findings shed light on the significant contributions of deep learning to text mining, information metrics, and scientific communication.

### 5.1 Deep learning for text mining

Text mining is discovering meaningful patterns and insights from unstructured text data. With the rapid growth of digital information, text mining has become increasingly important in many fields, such as social media analysis, customer reviews, and scientific literature mining. Deep learning has shown great potential in text mining, particularly in tasks such as natural language understanding, sentiment analysis, and topic modeling. In this study, we applied deep learning models to paragraph-level structure-function recognition and achieved promising results. The SciBERT model outperformed other models, highlighting the effectiveness of domain-specific pre-training in improving scientific literature performance. This finding is consistent with previous research and demonstrates the advantages of domain-specific pre-training in text classification tasks.

### 5.2 Deep learning for information metrics

Information metrics are methods and tools used to measure and analyze information. Information metrics can provide valuable insights into the distribution, impact, and quality of research outcomes in scientific literature. Deep learning has the potential to revolutionize information metrics by making the analysis of scientific literature more accurate and efficient. For example, deep learning models can identify the most influential papers or authors in a specific field, predict the future impact of research, and automate the literature review process. The results of this study demonstrate the enormous potential of deep learning models in identifying paragraph-level structural features in scientific literature, which can improve the efficiency of literature review and facilitate the discovery of new knowledge.

In addition, deep learning models can also be used for literature network analysis, such as co-citation analysis, co-cited document analysis, and citation visualization. These analyses can help researchers better understand the dynamics and trends in research fields. Furthermore, deep learning can be used for text generation tasks such as automatic summarization, machine translation, and knowledge graph generation, which can assist in producing, translating, and cross-disciplinary collaboration of scientific literature. Therefore, applying deep learning in scientific literature information metrics has a broad prospect and is expected to promote the development of scientific research and scholarly communication.

### 5.3 Deep learning for scientific communication

Deep learning can play a crucial role in facilitating scientific communication by enabling us to understand scientific literature better and thus engage in more effective communication. Deep learning models can automatically extract critical information, identify similar research, and construct knowledge graphs from a large amount of literature. These applications can help researchers quickly acquire knowledge, discover new research areas, and promote collaboration and interdisciplinary research. Furthermore, deep learning can aid in the translation of scientific literature across languages and assist in identifying and mitigating language barriers that impede global scientific communication. By leveraging the power of deep learning, we can overcome many obstacles that hinder scientific communication and promote sharing of knowledge and ideas across the scientific community.

## 6 Conclusion

In summary, our study demonstrates the potential of deep learning models for paragraph-level structure-function recognition in scientific literature. The SciBERT model outperformed other models in this task, highlighting the effectiveness of domain-specific pre-training for scientific literature. Deep learning has the potential to revolutionize text mining, information metrics, and scientific communication by enabling more accurate, efficient, and innovative analysis of scientific literature. Further research is needed to explore the full potential of deep learning in these areas and to develop new applications that can benefit researchers and society.

## Data availability statement

The raw data supporting the conclusions of this article will be made available by the authors, without undue reservation.

## Author contributions

JL: Methodology, Writing—original draft, Data curation, Writing—review & editing. ZZ: Data curation, Writing—review & editing. NW: Writing—original draft, Writing—review & editing. XW: Writing—original draft.
